# Systemic inflammation, innate immunity and pathogenesis after Zika virus infection in cynomolgus macaques are modulated by strain-specificity within the Asian lineage

**DOI:** 10.1080/22221751.2021.1943536

**Published:** 2021-06-14

**Authors:** Ruklanthi de Alwis, Raphaël M. Zellweger, Edmond Chua, Lin-Fa Wang, Tanu Chawla, October M. Sessions, Damien Marlier, John E. Connolly, Veronika von Messling, Danielle E. Anderson

**Affiliations:** a Programme in Emerging Infectious Diseases, Duke-NUS Medical School, Singapore; b Viral Research and Experimental Medicine Centre, SingHealth-Duke NUS, Singapore; c International Vaccine Institute, Seoul, South Korea; d Institute of Molecular and Cell Biology, A*STAR, Singapore; e Saw Swee Hock School of Public Health, National University of Singapore, Singapore; f Department of Pharmacy, National University of Singapore, Singapore; g Institute of Molecular and Cell Biology, Agency for Science, Technology and Research, Singapore; h Department of Microbiology and Immunology, Yong Loo Lin School of Medicine, National University of Singapore, Singapore; i Institute of Biomedical Studies, Baylor University, Waco, TX, USA; j Veterinary Medicine Division, Paul-Ehrlich-Institute, Langen, Germany; k Department of Microbiology and Immunology, University of Melbourne, Peter Doherty Institute for Infection and Immunity, Melbourne, Australia

**Keywords:** Zika virus, flavivirus, arbovirus, NHP, inflammation, innate immunity

## Abstract

Zika virus (ZIKV) is an emerging arbovirus with recent global expansion. Historically, ZIKV infections with Asian lineages have been associated with mild disease such as rash and fever. However, recent Asian sub-lineages have caused outbreaks in the South Pacific and Latin America with increased prevalence of neurological disorders in infants and adults. Asian sub-lineage differences may partially explain the range of disease severity observed. However, the effect of Asian sub-lineage differences on pathogenesis remains poorly characterized. Current study conducts a head-to-head comparison of three Asian sub-lineages that are representative of the circulating ancestral mild Asian strain (ZIKV-SG), the 2007 epidemic French Polynesian strain (ZIKV-FP), and the 2013 epidemic Brazil strain (ZIKV-Brazil) in adult Cynomolgus macaques. Animals infected intervenously or subcutaneously with either of the three clinical isolates showed sub-lineage-specific differences in viral pathogenesis, early innate immune responses and systemic inflammation. Despite the lack of neurological symptoms in infected animals, the epidemiologically neurotropic ZIKV sub-lineages (ZIKV-Brazil and/or ZIKV-FP) were associated with more sustained viral replication, higher systemic inflammation (i.e. higher levels of TNFα, MCP-1, IL15 and G-CSF) and greater percentage of CD14+ monocytes and dendritic cells in blood. Multidimensional analysis showed clustering of ZIKV-SG away from ZIKV-Brazil and ZIKV-FP, further confirming sub-lineage differences in the measured parameters. These findings highlight greater systemic inflammation and monocyte recruitment as possible risk factors of adult ZIKV disease observed during the 2007 FP and 2013 Brazil epidemics. Future studies should explore the use of anti-inflammatory therapeutics as early treatment to prevent ZIKV-associated disease in adults.

## Introduction

Zika virus (ZIKV) is a human pathogen that in the past decade has emerged as a virus of public health concern [1]. ZIKV belongs to the *Flaviviridae* family, which encompass several other viruses causing widespread human disease such as dengue virus (DENV), Japanese encephalitis virus (JEV), yellow fever virus (YFV) and West Nile virus (WNV) [2]. Although ZIKV is primarily spread through the *Aedes* mosquito vector, other means of transmission have also been reported [3,4]. Maternal-to-fetal vertical transmission of ZIKV through the placenta during pregnancy have been linked with increased risk of preterm birth, stillbirth, as well as a number of debilitating neurological congenital pathologies collectively grouped under the term congenital Zika syndrome (CZS) (which includes microcephaly, abnormal development of the brain, ocular pathologies, limb contractures and other neurologic abnormalities) [5–7,8]. Conversely, acute ZIKV infection in adults often remains unnoticed, and symptomatic cases are characterized by a mild flu-like disease, inluding fever, headaches, myalgia and arthralgia, and occasionally conjuncivitis, rash, and gastro-intestinal signs, with an onset at 3–12 days after exposure [9,10]. Although ZIKV is rarely associated with mortality or severe acute disease, based on epidemiological data ZIKV infections have also been linked with rare neurological diseases and autoimmune Guillain-Barré Syndrome (GBS) [5,6,11,12].

ZIKV was first isolated from a sentinel rhesus macaque in the Zika forest in Uganda in 1947 [13]. The following year, the virus was isolated from *Aedes africanus* mosquitoes [13], and the first mild febrile human cases due to ZIKV were soon documented in the same region [14]. In 1966, the presence of ZIKV in Asia was confirmed as the virus was isolated from *Aedes aegypti* mosquitoes in Malaysia [15]. For the next 60 years, rare human ZIKV cases were mainly restricted to Africa and Asia, with disease reported as being generally mild or asymptomatic [16]. However, the geographical distribution of ZIKV dramatically changed with the outbreak in Micronesia in 2007 [17], which marked the beginning of ZIKV expansion across the Pacific and beyond. In 2013, an outbreak started in French Polynesia and spread to multiple Pacific Islands, where ZIKV infections were linked to neurological complications in adults [18]. Subsequently, in 2015, ZIKV reached South America, with Brazil, Colombia and Venezuela reporting the majority of cases [18]. Interestingly, this geographical expansion of ZIKV into the Pacific Islands and then South America was directly or indirectly associated with an increase in neurological pathologies, most notably CZS in infants [12] and auto-immune pathology such as Guillain-Barré syndrome (GBS) in adults [11].

ZIKV has now been reported in 86 countries and territories according to the world health organization (WHO) [19]. However, ZIKV-associated neurological complications seem to be mainly restricted to the Pacific and the Americas [8]. Microcephaly and GBS were primarily observed in Brazil and French Polynesia, but much less reported in Southeast Asia [20,21]. Only 3 cases of ZIKV-related microcephaly have been reported in Thailand, and 1 in Vietnam [22,23]. Also, the 2016 epidemic of ZIKV in Singapore did not cause a surge in the number of GBS in adults, unlike what was observed in Polynesia or South America [24]. Similarly, no ZIKV-related CZS in infant cases were reported in Singapore during the 2016 ZIKV outbreak [25]. The apparent low rate of ZIKV-associated neurological complications in Southeast Asia has several possible explanations, including the relatively small size of well-documented ZIKV outbreaks, or the overall low reporting rate for ZIKV and microcephaly in Southeast Asia [21,22,24,25]. Another plausible explanation is the presence of viral determinants [1,22,26], since the Asian lineage is composed of three sub-lineages corresponding to the geographical areas of spread (i.e. the Southeast Asian, the Pacific and the American sub-lineages [27,28]).

Unlike the effects of inter-lineage differences (Asian versuses African lineages), which have been abundantly investigated [26,29–32], much less is known about the impact of sub-lineage differences on ZIKV pathogenesis and early host immune responses. To address this knowledge gap and assess the impact of the ZIKV Asian sub-lineages on ZIKV infection in adults, we perfomed a systematic head-to-head comparison of viral pathogenesis and early immune responses in adult cynomolgus macaques following infection with clinical isolates from Singapore, French Polynesia and Brazil. These three strains were chosen as they represent the three sub-lineages defined within the Asian lineage, namely Southeast Asian, Pacific and American.

## Materials and methods

### Cells and viruses

Baby hamster kidney (BHK-21) cells (ATCC #CCL-10) and C636 (ATCC #CRL-1660) were maintained in RPMI medium supplemented with 8% foetal bovine serum (FBS) and 10 mM Hepes. ZIKV strains ZIKV-Brazil (strain BeH815744, GenBank ID: KU365780.1), ZIKV-FP (strain PF13/251013–18, GenBank ID: KX369547) and ZIKV-SG (strain ZKA-16-291, GenBank ID: KX827309.1) were amplified on C6/36 cells. The full-genome of each virus was sequenced on an Illumina MiSeq upon acquisition to confirm identity. Virus titres were quantified by plaque assay and expressed as plaque forming units per millilitre (pfu/ml).

### Plaque assay

All ZIKV strains were titrated on BHK-21 cells. Ten-fold serial dilutions of virus culture supernatant were added to monolayer of BHK-21 cells in 24-well plates and incubated for 1 h at 37°C with gentle rocking every 15 min. The inoculum was aspirated, replaced with 1% methyl-cellulose in maintenance medium (RPMI-1640, 2% FCS, 10 mM Hepes, penicillin, and streptomycin) and incubated at 37°C. At 5 days post-infection (dpi), cells were fixed with 4% formaldehyde at room temperature for 20–30 min, washed with H_2_O, and stained with 1% crystal violet for 20 min. The plates were washed, dried, and the titre was calculated and expressed as pfu/ml.

### Animal experiments and assessment of virulence

Cynomolgus macaques (*Macaca fascicularis*) were used for all studies. The experiments were approved by the SingHealth Institutional Animal Care and Use Committee of the SingHealth Experimental Medicine Centre (SEMC). Cynomolgus macaques were purchased from the SingHealth colony and free of antibodies against DENV and ZIKV. Animals were infected via intervenous (IV: ZIKV-Brazil *n*=3, ZIKV-FP *n*=3, ZIKV-SG *n*=3) or subcutaneous (SC: ZIKV-Brazil *n*=6, ZIKV-FP *n*=3, ZIKV-SG *n*=3) routes with 5 × 10^6^ pfu of the respective virus. Passage 2 stocks were used for the NHP studies. Post-infection, animals were observed daily for activity and rash. Daily body temperature was monitored using an orally applied temperature sensor (Anipill, DSI) and rectally whenever the animals were anesthetized. Ingestable temperature sensors have previously been validated in humans [33]. Anipill swallowable capsule continuously measures core body temperature data and the sensor was programmed to collect a measurement every 15 min. At days 0–5, 7, 9, and 11 post-infection, the animals were anesthetized with ketamin, weighed, throat swabs collected and blood samples collected from the femoral vein. At day 11, 18 (out of 21 animals) were euthanized, and samples were collected for the isolation of RNA and immune cells.

### RNA purification and in vitro transcription

RNA was isolated from serum samples using ZR Viral RNA (Zymo Research) according to the manufacturer’s instructions. Tissue samples were weighed and RNA was extracted from 30 mg of tissue using the E.Z.N.A. Total RNA Kit I (Omega Biotek) according to the manufacturer’s instructions. Templates for *in vitro* transcription were generated by amplifying ZIKV cDNA by PCR using a forward primer tagged with a T7 promoter; ZIKV-T7-F: 5'- TAA TAC GAC TCA CTA TAG GCT CCC CTC CCA TTC-3’. The fragment was reverse transcribed using HiScribe T7 High Yield RNA Synthesis Kit (NEB). DNA was removed by DNase treatment with DNase I (NEB) for 15 min at 37°C and the RNA purified using RNeasy mini kit (Qiagen). The total amount of RNA was quantified using a Nanodrop (ThermoScientific). The number of RNA molecules produced by *in vitro* transcription was calculated using the following formula: RNA molecules/µL = [µg RNA / transcript length x 340] x [6.022 × 10^23^].

### Quantitative real-time PCR

Quantitative PCR (qPCR) was performed using QuantiTect Probe RT–PCR Kit (Qiagen) reagents and with the CFX96 Real-Time System (Bio-Rad). Each 25 µL PCR reaction contained 12.5 µL 2X QuantiTect PCR mastermix, 1 µL of each 10 mM primer, 0.5 μL 0.2 mM probe, 0.5 μL reverse transcriptase, 3 µL RNA template and 6.5 µL H_2_O. Every PCR was performed as follows: initial PCR activation at 95°C for 5 min and 45 amplification cycles consisting of a 95°C denaturation for 10 sec and a 60°C annealing/extension for 30 sec. Sequences of primers and probes used in the presnt study are 100% conserved across the three ZIKV strains and are as follows; ZIKV-F: 5'- TGG TCA TGA TAC TGC TGA TTG C -3’, ZIKV-R; 5'- CCT TCC ACA AAG TCC CTA TTG C -3’, ZIKV-probe5'- /56-FAM/CGG CAT ACA GCA TCA GGT GCA TAG GAG /3BHQ_1/ -3’. Each PCR was done in duplicate. Amplicons were quantified by plotting the Ct values against standard curves made using 10-fold dilutions of cDNA produced from *in vitro* transcription RNA samples. Data were expressed as molecules of RNA.

### Antibody responses

Blood samples were collected in to SST tubes, allowed to clot at room temperature for 1 h, and centrifuged at 3000 rpm for 15 min. Serum aliquoted and stored at −80°C until use. IgM and IgG in serum samples were quantified using an in-house ELISA. Briefly, sucrose-purified ZIKV-Brazil (50 ng/well) was coated on high binding ELISA plates (Maxisorp, NUNC) at 4°C overnight, then washed with 0.2% Tween-20 in 1x PBS and blocked with 1% casein block for 1 h at 37°C. Serum samples were then diluted 1:100 in dilution buffer (0.1% Tween-20 in blocking buffer) and incubated on ELISA plates for 1 hr at 37°C. Plates were then washed and probed for IgM, IgA and IgG using anti-monkey IgM-HRP (Novus Biologicals, NBP1–73726), anti-monkey IgA-HRP (Sigma, SAB3700759) and anti-monkey IgG-HRP (Santa Cruz Biotec, Sc-2458), respectively.

### Assessment of innate immune cell populations

PBMCs were isolated from NHP whole blood using Ficoll gradients, according to manufacturer’s instructions. Plasma was stored at −80°C until further use. PBMCs were stained for monocytes and dendritic cells using the following antibody panel, CD16-BV605, CD14-PE-Cy7, CD11c-PE, HLA-DR-BV786, CD123-PE-CF594, CD20-BV711, CD159a-APC, Live/Dead-APC-Cy7 (BDbiosciences). Following a 30 min incubation with the antibody master mix, PBMCs were washed with staining buffer [1x PBS (Invitrogen), 2% FCS (Hyclone), 2 mM EDTA (Invitrogen), 15 mM HEPES (Invitrogen)] and analysed on BD LSR II.

### Assessment of plasma cytokines

Cytokines (i.e. IFNγ, TNFα, MCP-1, IL-15, IL-10, IL1RA, MIP-1β, IL-6, IL-1β, IL-12p40 and G-CSF) in the plasma was assessed using the Non-Human Primate Cytokine/Chemokine Panel 1 (Merck Milliplex #PRCYTOMAG-40K) kit and by following manufacturer’s protocol. Briefly, plasma samples collected at various timepoints were incubated with capture beads in the kit, then captured cytokines detected using detection antibodies from the kit, and read with the MAGPIX instrument using xPONENT software. Median fluorescent intensity (MFI) from beads with plasma samples were compared against a standard curve to estimate respective cytokine concentrations in the plasma samples.

### Phylogenetic analysis

**Figure 1.**
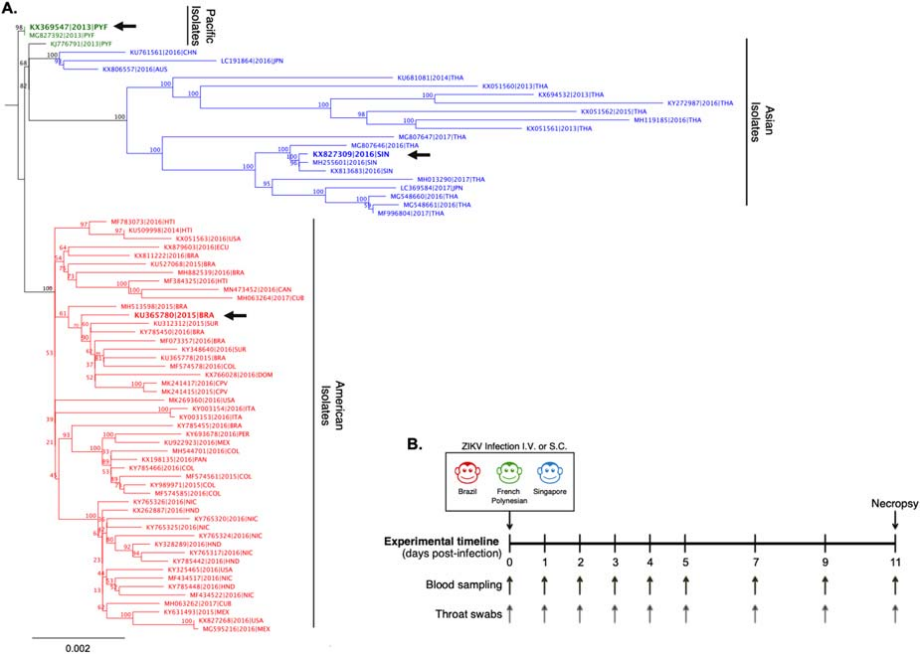
Phylogenetic comparison and experimental study design comparing clinical isolates of ZIKV from Singapore, French Polynesia and Brazil. **A**) Phylogenetic analysis of ZIKV full-length genomes isolated from the Pacific region, Asia and the Americas. The ZIKV isolates used in the present study from Brazil (ZIKV-Brazil), French Polynesia (ZIKV-FP), and Singapore (ZIKV-SG) are marked on the phylogenetic tree with a black arrow. **B**) Study timeline of infection of cynomolgus macaques with ZIKV. Animals were infected either IV or SC with either ZIKV-Brazil, ZIKV-FP or ZIKV-SG (5 × 10^6^ pfu/animal). Blood and throat swab samples were collected from animals at baseline, 1–5, 7, 9 and 11 dpi. Animals were euthanized and necropsied for tissues and organs on 11 dpi. Sample sizes for IV infections consisted of ZIKV-Brazil *n*=3, ZIKV-FP *n*=3, and ZIKV-SG *n*=3, and SC infections consisted of ZIKV-Brazil *n*=6, ZIKV-FP *n*=3, and ZIKV-SG *n*=3.

All complete genome sequences of ZIKV isolates (476 genomes) available on NCBI at the time of analysis were aligned using MAFFT v7.309. Initial topographical tree was built with FastTree v2.1.11 with a GTR model of substitution. Redundancy and outliers were removed and remaining genomes were re-aligned using RAxML v7.2.8 (starting with a complete random tree, then 1000 bootstraps and GTR-GAMMA model of substitution) to build a more precise maximum likelihood tree. The final phylogenetic tree (presented in Figure 1) was build using 72 full ZIKV genomes that included the ZIKV strains (ZIKV-FP, ZIKV-Brazil and ZIKV-SG) used in the current animal study.

### Data analysis

Multidimensional unsupervised analyses, hierarchical clustering and principal component analyses (PCA), were conducted using AUC of the following variables over time - throat viremia (ZK throat), serum viremia (ZK serum), % of monocytic cells (Mon), % of CD14^+^CD16^-^ monoctyes (cMon), % of CD14^+^CD16^+^ monoctyes (CD16+ Mon), plasmacytoid DCs (pDC), myeloid DCs (mDC), IFNγ, TNFα, MCP-1, IL-15, IL-10, IL1RA, MIP-1β, IL-6, IL-1β, IL-12p40, G-CSF, IgM, IgA, and IgG. Unsupervised hierarchical clustering was conducted using the R package pheatmap [34]. Original values were ln(x + 1)-transformed, rows were centred and scaled, and both rows and columns were clustered using Manhattan distance and complete linkage. Principal component analysis (PCA) was performed to describe the multivariate immunological profiles using R packages FactoMineR [35] and factoextra [36]. The individuals on the individual plot were color-marked according to the route of infection, or according to both ZIKV strain and route of infection.

Statistical differences across groups at different time points were compared using 2-way ANOVA and Turkey’s multiple comparison test. Statistical comparison of AUC between groups was conducted using unadjusted Wilcoxon test. All calculations were performed either using GraphPad Prism v8 Software or R software v3.5.1 [37].

## Results

### Clinical features following infection with ZIKV strains from different geographic regions

**Figure 2.**
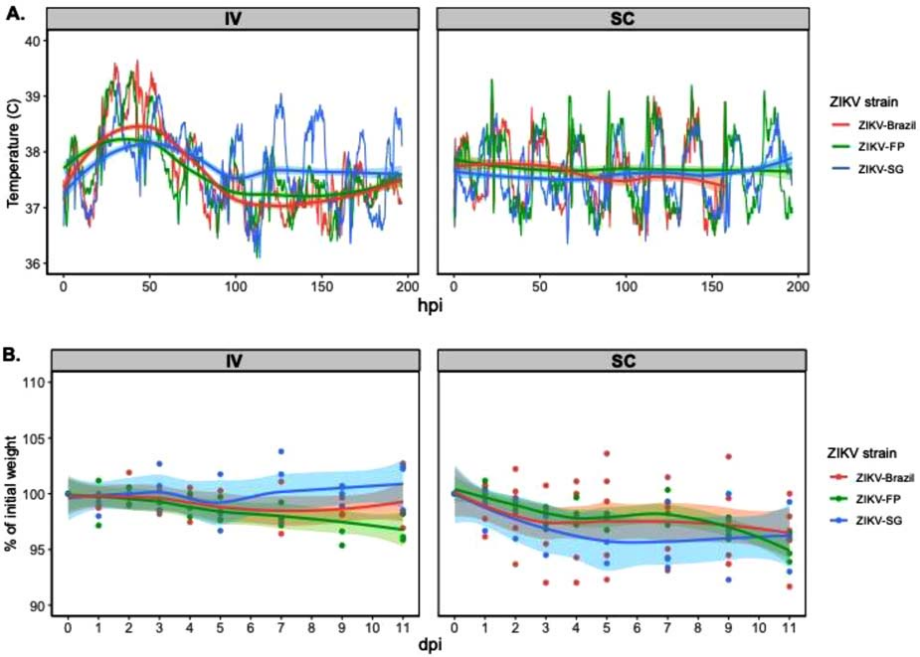
Body temperature and weight change in cynomolgus macaques infected with ZIKV. **A**) Body temperature (°C) in monkeys after IV or SC infection with ZIKV. Average body temperatures for each ZIKV group graphed before and after Loess smoothing. **B**) Changes in body weight (% of initial weight) after IV or SC infection with ZIKV (displayed as individual values and as average Loess smoothed curves with 95% CI in grey).

To compare the pathogensis and early immune responses between the ZIKV Asian sub-lineages (i.e. Southeast Asian, the Pacific and the American), we chose clinical isolates that were isloated from Singapore in 2016 (ZIKV-SG), French Polynesia in 2013 (ZIKV-FP) and Brazil in 2015 (ZIKV-Brazil) (as shown in Figure 1A). Since past ZIKV studies in NHPs have utilized various routes of infection and it is yet unclear which route best mimics infection through a mosquito bite [38–41], we further compared responses of the different ZIKV sub-lineages when infected through either a IV or SC route. Three to six adult cynomolgus macaques were infected with 5 × 10^6^ pfu of either ZIKV-Brazil, ZIKV-FP, or ZIKV-SG via the intravenous (IV) or subcutaneous (SC) routes (Figure 1B). Clinical features were assessed following ZIKV infection, with highly detailed body temperature recordings (every 15 min) also capturing changes due to circadian rhythm. Animals infected with ZIKV through the IV route experienced sustained fever reaching temperatures of above 39°C for the first two days post-infection (Figure 2A), while no sustained fever was detected in any of the animals infected through the SC route. No differences in the average animal body temperatures was detected when comparing groups of animals infected with either ZIKV-Brazil, ZIKV-FP or ZIKV-SG until 100 h post infection, but temperature was slightly elevated in ZIKV-SG infected animals thereafter (Figure 2A). Similarly, no changes in weight were observed during the study period regardless of the ZIKV isolate within each route of infection. However, slight weight loss was observed in animals infected SC (regardless of the isolate), but not in the IV infected animals. (Figure 2B). No additional clinical signs such as rash, eye redness, loss of appetite, diarrhea, dehydration, depression or inactivity were observed throughout the experimental period were observed. Our data are consistent with findings reported from other primate studies, where no major changes were observed in clinical signs or symptoms post-ZIKV infection in NHPs [40–42].

### ZIKV sub-lineage-specific differences in viremia detected in the mucosa

**Figure 3.**
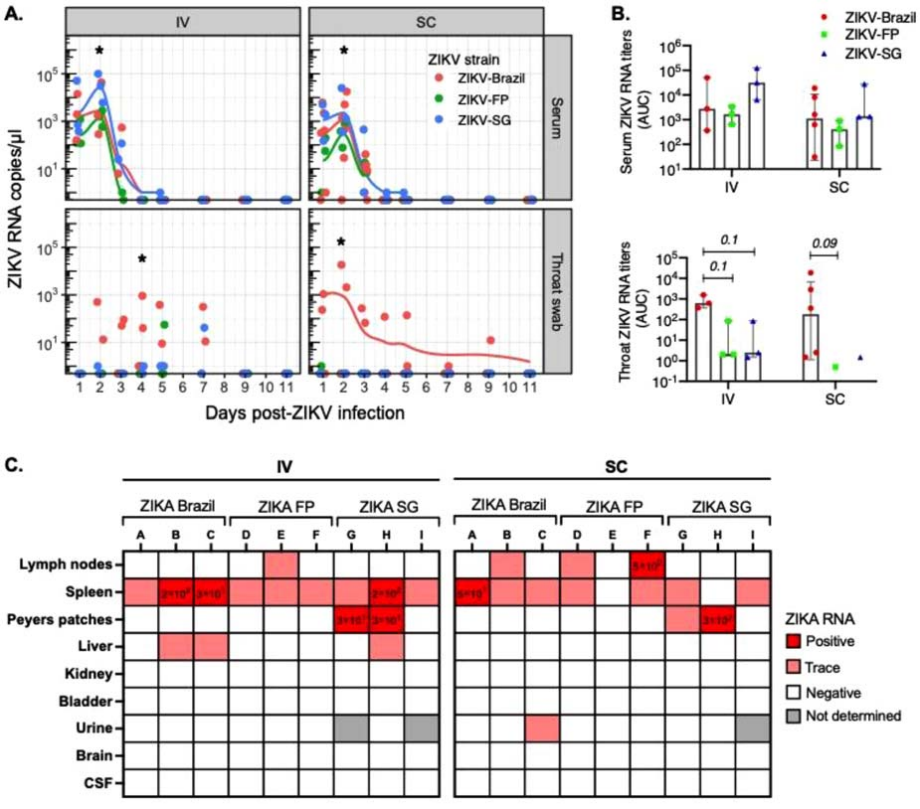
Viral kinetics in serum, throat swabs and organs after ZIKV infection. Longer ZIKV viral presence was detected in throat swabs of ZIKV-Brazil infected animals. **A**) ZIKV RNA titres were assessed at 1–5, 7, 9 and 11 dpi in serum and throat swabs of cynomolgus macaques infected with ZIKV-Brazil, FP or SG by either IV or SC. **B**) ZIKV RNA titres over time in serum and throat swab samples was assessed for each group as area under the curve (AUC). AUC are graphed as median and inter-quartile range (IQR). **C**) Viral RNA titres in organs were assessed following euthanasia of macaques on day 11 post-infection. ZIKV RNA quantity for samples that were within the standard curve are labelled under “Positive” with RNA quantity displayed (as copies/ μl). Samples with a detectable signal but below the standard curve are labelled as “Trace”. Samples with no detectable signal are labelled as “Negative”, and samples that were unavailable for PCR quantification are labelled as “Not determined”. Tissue viral data are presented for all study animals, except three macaques in the ZIKV-Brazil group that were not euthanized on day 11 post-infection. Statistical differences between groups at each time point was calculated using two-way ANOVA adjusted for Turkey’s multiple comparison (*p<0.05). Statistical comparison of AUC between groups was conducted using unadjusted Wilcoxon test (*p<0.05).

Serum viremia is a hallmark of flavivirus infection, and levels around 10^4^–10^5^ copies/ml are reported in ZIKV-infected patients [43,44]. In NHPs, regardless of route of ZIKV infection or sub-lineage, serum viral RNA titres peaked at 2 days post-infection and then rapidly decreased to undetectable levels by day 5–7 post-infection (Figure 3A). In general, the serum viremia peak at 2 days post-infection was higher following IV infection as compared to SC. We then compared ZIKV sub-lineage-specific serum viremia, and found no statistical differences in the AUC of viral RNA titres over time (Figure 3B). However, we did observe that the peak viral RNA titres (at day 2 post-infection) were significantly higher in animals infected with ZIKV-SG than in ZIKV-Brazil or ZIKV-FP (Figure 3A).

As ZIKV has also been found in mucosal swabs and urine of patients [45,46], we investigated viral load kinetics in throat swabs and urine. Interestingly, viral RNA was predominantly detected in throat swabs of animals infected with ZIKV-Brazil, with viral loads in some ZIKV-Brazil-infected animals reaching levels as high as that detected in the serum (Figure 3A). Two IV and one SC-infected animals remained positive until day 7 or the end of the study on day 11, respectively, suggesting that the ZIKV-Brazil may have a tendency to disseminate to and persist at mucosal tissues. Assessment of organ viral load at necropsy detected no viral RNA in the brain or cerebrospinal fluid (CSF) samples, but detected viral RNA in the spleens of all but two animals, the liver of two ZIKV-Brazil, the lymph nodes of half of the ZIKV-FP, and the Peyer’s patches of several ZIKV-SG-infected animals (Figure 3C). Data shows lower viral RNA levels in the throat after infection with ZIKV from Asian sub-lineages. However, future studies with larger sample size of animals will have to be conducted to further explore sub-lineage-specific differences in tissue tropism.

### ZIKV sub-lineage-specific differences in cytokine responses

**Figure 4.**
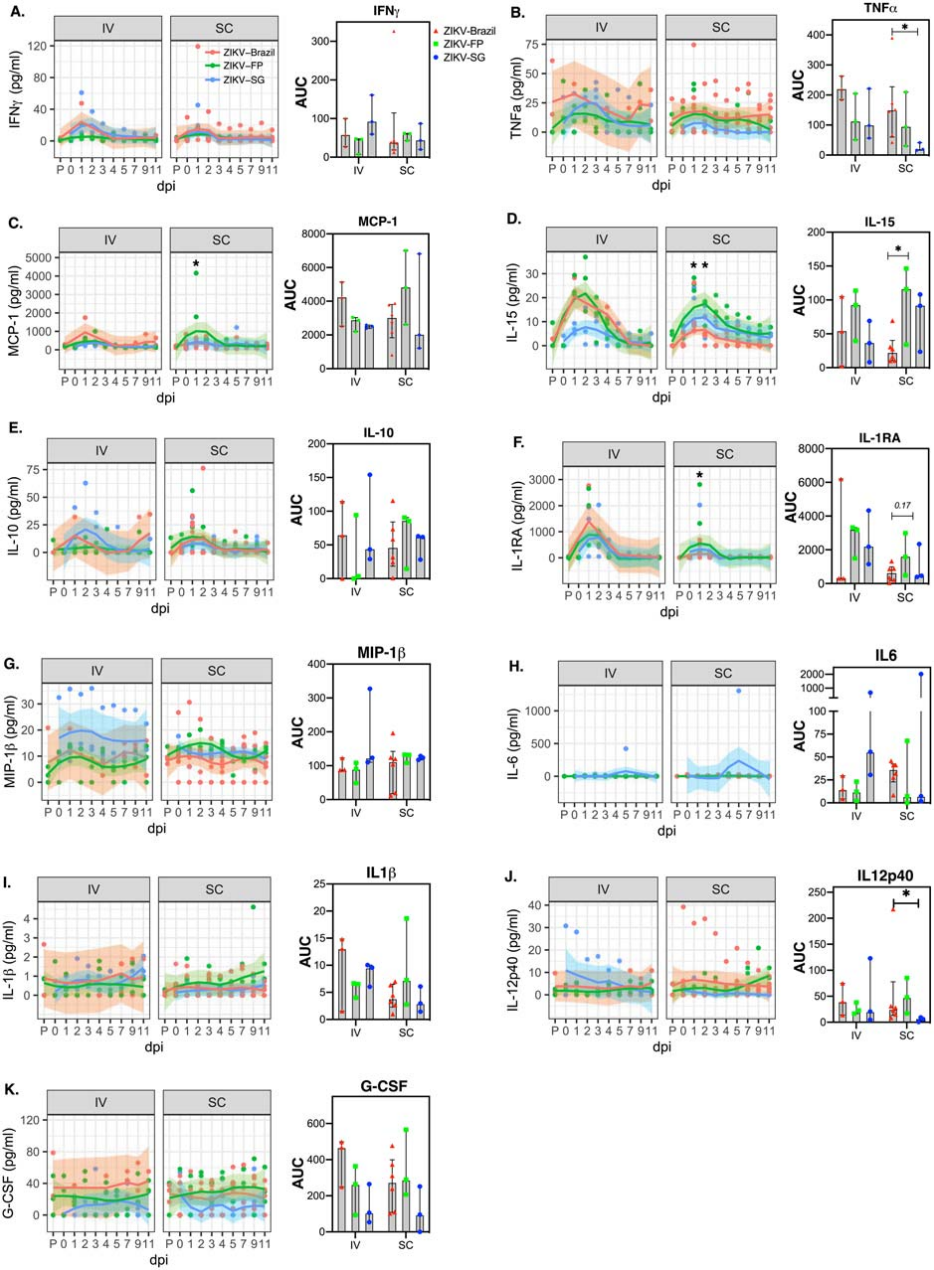
Kinetics of pro and anti-inflammatory cytokines following infection with different ZIKV isolates. The cytokines IFNγ (**A**), TNFα (**B**), MCP-1 (**C**), IL-15 (**D**), IL-10 (**E**), IL1RA (**F**), MIP-1β (**G**), IL-6 (**H**), IL-1β (**I**), IL-12p40 (**J**) and G-CSF (**K**) in plasma were assessed at baseline, and 1–5,7,9 and 11 dpi. Corresponding AUC was estimated for the measured cytokines over time (graphed as median and IQR). Cytokine curves over time was drawn using Loess smoothing with 95% CI represented in grey. AUC were graphed as median and inter-quartile range (IQR). At each time point statistical comparisons between groups was conducted using two-way ANOVA adjusted for Turkey’s multiple comparison (*p<0.05). Wilcoxon test was used to compare AUC between groups (*p<0.05)

To evaluate the interactions between the different ZIKV sub-lineages and the host innate immune system, we first investigated the kinetics of pro and anti-inflammatory cytokines and chemokines in the serum. In general, ZIKV infection elicited rapid increase in serum cytokines and chemokines (specifically IFNγ, TNFα, MCP-1, IL-15, IL-10, IL1RA, MIP-1β (Figure 4A-G)), with responses peaking around 1–2 days post-infection. Not surprisingly, ZIKV infection through the IV route elicited higher peak cytokine and chemokine responses than SC infection. Due to the small sample size and the large variation, ZIKV sub-lineage-specific differences in the cytokine and chemokine responses were unclear. However, it was noted that the highest production of TNFα, MCP-1, IL15 and G-CSF were associated with ZIKV-Brazil and/or ZIKV-FP, suggesting potentially greater inflammatory responses elicited by these two sub-lineages in comparison to ZIKV-SG (Figure 4).

### Greater recruitment of innate immune cells by ZIKV isolates from Brazil and FP

**Figure 5.**
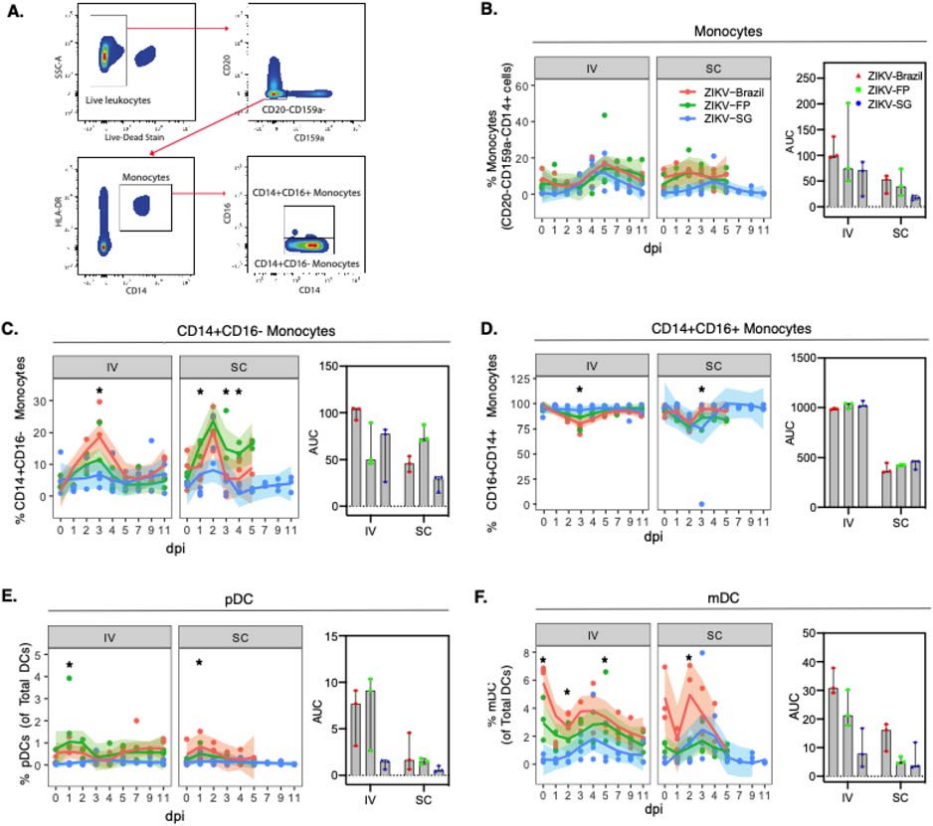
Monocytes and dendritic cells in the blood following ZIKV infection. **A**) The gating strategy of monocytes and dendritic cells, **B**) frequency and AUC of monocytic cells (CD20-CD159a-HLA-DR + CD14+), **C-D**) monocytic cell subsets (CD14+CD16- monocytes and CD14+CD16+ monocytes), **E**) plasmacytoid DCs (pDC) and **F**) myeloid DCs (mDC) were assessed in PBMCs. Data for time points 7, 9 and 11 dpi following SC infection with ZIKV-Brazil and ZIKV-FP is unavailable, therefore for SC AUC was only computed for the time period days 0–5. Curves of immune cell percentages over time were drawn using Loess smoothing with 95% CI of the smoothed curve represented in grey. AUC were graphed as median and inter-quartile range (IQR). Two-way ANOVA adjusted for Turkey’s multiple comparison (*p<0.05) was conducted at each timepoint, and Wilcoxon tests between groups for AUC (*p<0.05).

Published literature has characterized ZIKV infection as highly inflammatory with rapid recruitment of innate immune cells [47]. Therefore, we also investigated the effect of ZIKV infection on the circulating blood monocyte and dendritic cell (DC) populations (Figure 5A). Following ZIKV infection, higher percentage of monocytes (CD20^-^CD159a^-^CD14^+^cells) were detected in the blood after infection, with similar responses across the different ZIKV sub-lineages (Figure 5B). Further dissection of the monocyte populate into subpopulations showed that the percentage of classical monocytes (CD14^+^CD16^-^) also rapidly increased in the cirulating blood and peaked around 2–3 days post-ZIKV infection. When we compared responses across the ZIKV sub-lineages, we found that ZIKV-Brazil and ZIKV-FP elicited significantly greater percentage of classical monocyte population at 3 days post-infection in IV and 1,3,4 days post-infection in SC (Figure 5C). It’s been shown that non-classical monocytes are a major target of ZIKV infection [47], therefore we also investigated the kinetics of the non-classical monocyte population. The percentage of non-classical monoctyes (CD14^+^CD16^+^) decreased following ZIKV infection with the lowest point reaching around 2–3 days post-infection (Figure 5D). Differences in the percentage of non-classical monoctyes between ZIKV isolates were observed 3 days after IV infection, where ZIKV-Brazil elicited the lowest percentage of non-classical monoctyes.

DCs are professional antigen presenting cells (APCs) that have previously been shown to increase in frequency in the blood in response to ZIKV infection [47,48]. Therefore, we then characterized the DC populations, plasmacytoid DCs (pDCs) and myeloid DCs (mDCs). Following ZKV infection, we observed rapid increase in the frequency of both pDCs and mDCs in circulation with peaks at 1–2 days and 3–4 days post-infection, respectively (Figure 5E and F). The frequency of pDCs and mDCs (at peak and AUC) were both significantly greater in ZIKV-Brazil and ZIKV-FP than ZIKV-SG-infected animals (Figure 5E and F), further confirming the more inflammatory nature of the ZIKV-Brazil and ZIKV-FP sub-lineages. Unsurprisingly, IV infection of ZIKV elicited in general greater innate immune cell responses than SC infection (as displayed by the AUC assessment of percentage monocytes, classical monocytes, non-classical monocytes, pDCs and mDCs) (Figure 5).

### No ZIKV sub-lineage-specific differences in early antibody responses

**Figure 6.**
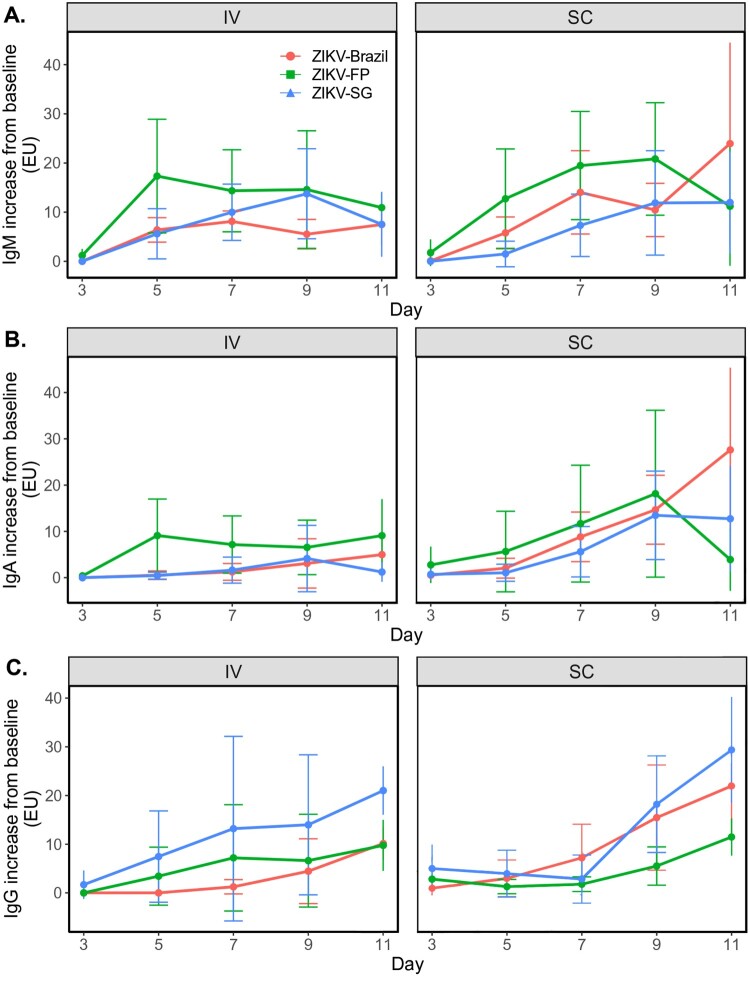
Early antibody responses following infection with ZIKV isolates. Increase in antibodies from baseline in all experimentally infected animals was assessed for IgM (**A**), IgA (**B**) and IgG (**C**) at days 3, 5, 7, 9 and 11 dpi. Antibody data at each timepoint graphed per ZIKV group (mean ± SD).

In naïve individuals, early antibody responses correlate with virus control and clearance, while delayed and dysfunctional antibody responses are often associated with exacerbated disease [49–51]. As expected, ZIKV-specific IgM responses were detected soon after infection with no major differences in kinetics between route of infection or ZIKV isolates (Figure 6A). ZIKV IgA and IgG response kinetics were slower but gradually increased over the course of the study, with antibody titres still rising at day 11 post-infection (Figure 6B and C). No overall differences in IgA and IgG kinetics were detected between the three tested ZIKV sub-lineages. However, longer term studies with virus neutralization assessment will have to be conducted to truly assess the effect of different ZIKV Asian sub-lineages on antibody responses.

### Unsupervised multidimensional analyses reveal route- and strain-specific virological and inflammatory signatures

**Figure 7.**
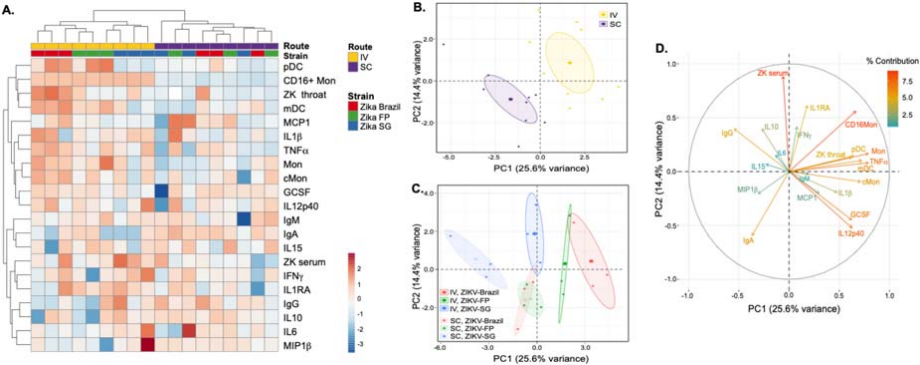
Unsupervised clustering analysis display clustering by route of infection and by ZIKV strain. **A**) Hierarchical clustering was conducted using Manhattan distance and complete linkage to all rows and columns. PCA was conducted on all measured viremia, cytokine (AUC), immune cell subsets (AUC) and antibody responses (AUC), with 95% confidence ellipses indicating the different **B**) routes of infection or **C**) ZIKV strains. **D**) The circle plot indicates the variables contributing to the spread of points in the PCA plots.

Multidimensional analysis integrating immune responses and virological profiles after infection were performed to uncover potential patterns specific to the route of infection (IV vs SC) or the viral strain used for infection. AUC of the following variables, namely viral load in throat and serum, cytokine expression, cellular responses and antibody titres and were included in hierarchical clustering and PCA. During hierarchical unsupervised clustering, the IV and SC routes of infection clustered separately, indicating that distinct routes of infection induce distinct inflammatory and virological profiles (Figure 7A). Within the IV route of infection, ZIKV-Brazil and ZIKV-FP than ZIKV-SG also clustered separately, suggesting distinct signatures. This was not the case after SC infection, where no clear clustering of the three strains was observed.

PCA also confirmed distinct patterns in immune and virological parameters after infection when animals were coloured by route of infection (Figure 7B) or by viral strain (Figure 7C). On the plane defined by the first 2 components of the PCA, data points representing animals infected IV were mostly located in the upper right of the plane, clearly separated from the SC-infected animals, which were mostly located in the lower left corner. The circle plots (Figure 7D) describes the relationship between the variables, and their contribution to the spread of points. Based on the circle plot, IV-infected animals typically had higher values for serum and throat ZIKV viral titres, TNFα, IL1RA and percentage of DCs and monocytes (to mention a few variables contributing highly to the spread of points in the PCA), while SC-infected animals had higher values for IgA. Similar trends can also be observed on the heatmap in Figure 7A and in the correlation analysis between ZIKV RNA titres, cytokine and immune cell parameters (Supplementary Figure 1).

When animals were coloured by strain and route of infection (Figure 7C), PCA displayed overlapping ellipses for ZIKV-Brazil and ZIKV-FP (in both SC and IV), while the 95% CI ellipse for ZIKV-SG was distinctively seperated. Differences between the ZIKV sub-lineages were primarily due to differences in the variables in component 1 (PC1) of the PCA. Infection with ZIKV-Brazil or ZIKV-FP as compared to ZIKV-SG was typically characterized by higher values of variables contributing to PC1, such as throat viremia, TNFα or the percentage of DCs and monocytes Figure 7C. This suggests that as opposed to ZIKV-SG, infection with the ZIKV-Brazil and ZIKV-FP isolates induces higher throat viremia and a higher inflammatory state. Overall, the multidimensional analysis suggests route- and strain-dependent modulation of inflammatory and virological responses after ZIKV infection in NHPs.

## Discussion

Unlike the ancestral ZIKV strains circulating in Asia, the 2007 and 2015 ZIKV epidemics in French Polynesia and the Americas (including Brazil) were caused by viral strains that were associated with significant neurological disease in humans. The current study investigated the Asian sub-lineage differences of ZIKV pathogenesis in an adult NHP model using clinical isolates from Singapore, French Polynesia and Brazil.

Following infection, despite no major sub-lineage-specific differences in body temperature and weight, Brazil isolates showed more sustained viral infection as detected by throat swabs. due to, We were unable to detect viral RNA in brain or CSF at the time of necropsy (d11 post-infection), possibly because of the long time interval between ZIKV infection and necropsy. Although not statistically significant, we observed that the early appearance of several pro-inflammatory cytokines and chemokines (TNFα, MCP-1, IL15, and G-CSF) were more associated with ZIKV-Brazil and/or ZIKV-FP infections. Greater systemic inflammatory responses in ZIKV-Brazil and ZIKV-FP isolates were further highlighted by the higher percentages of monocytes (particularly CD14+CD16- monocytes), pDCs and mDCs in blood. Multidimensional data integration and analysis (using unsupervised clustering techniques) graphically portrayed that both the route of infection and the ZIKV sub-lineage influenced pathogenesis, with IV infection and ZIKV-Brazil and ZIKV-FP isolates eliciting greater inflammatory host responses.

Sub-lineage differences in ZIKV replication has been observed in mammalian cells (*in vitro*) and in various organs (including brain and spinal cord) in IFNaR1 KO mice [52]. These differences in replication may result from genetic differences that exist between the Asian sub-lineages [53]. Sub-lineage specific genetic variation is highest in the PrM/M and NS1 genes of ZIKV [53]. Interesingly, structural genes have also been shown to play a role in increased neurovirulence in strains of African lineage in comparison to strains from the Asian lineage [31]. Our study did not observe major differences in ZIKV RNA in the brain or other organs potentially because the virus had already been cleared when the organs were assessed for viral RNA at 11 dpi. However, we did observe sub-lineage differences in the kinetics of viral infection, with the greatest quantity and persistance of virus being detected in the saliva of ZIKV-Brazil infected animals.

ZIKV strains from both the African and Asian lineages primarily target CD14+ monocytes (and some dendritic cells), which often leads to monocyte-associated inflammation [54–59]. In the current study, the ZIKV-BRAZIL lineage not only showed longer persistance of virus, but also greater monoctye (both CD14+CD16- and CD14+CD16+ monocytes) and dendritic cell recruitment and increased expression of certain pro-inflmmatory cytokines and chemokines. It is not surprising that longer duration of acute ZIKV infections can lead to greater inflammation and thereby, increased pathology. Studies in mouse models have shown that long-term persistance of ZIKV in the brain can lead to inflammation and increased risk of neuropathology [60]. Epidemiological studies in ZIKV infected patients with detectable viremia have demonstrated significantly higher levels of monocyte-associated chemokines such as MCP-1 and IP-10 [56]. Interestingly, even within infected patients, several early proinflmmatory cytokines (such as G-CSF, TNFα, IFNγ, IL17, IL-1β and MCP-1) closely mirrored trends in viremia, indicating a direct link between viral replication and inflammation [57]. Furthermore, inflammation resulting from ZIKV infections can lead to increased pathology, such as fetal growth malformations observed in ZIKV-infected pregnant mothers with high levels levels of IL22, MCP-1, TNFa, IP10 [55]. Similarly, a negative correlation was observed between systemic inflammation and the cephalic perimeter in newborns with ZIKV-associated microcephaly [58]. Therefore, systemic inflammation may also provide an explanation for the sub-lineage differences observed in the appearance of severe neurological disease.

In conclusion, the present study is the first head-to-head comparison in adult NHP of Asian sub-lineages that are epidemiologically associated with neurological disorders in humans (ZIKV-Brazil and ZIKV-FP) or not (ZIKV-SG). Particular strengths of this study is that it allowed observation of sub-lineage-specific responses in isolation of potential confounding factors, and sub-lineage differences were observed even in wild-caught, outbread NHPs which is more representative than inbread (genetically identical) mice. However, limitations exist in the small sample sizes per group, detection of virus only through viral RNA, virus titration in organs at a late timepoint of 11 dpi, and the lack of major clinical disease observed. Despite the limitations, our investigations observed that infections with ZIKV-Brazil lead to sustained viral infection leading to greater monocyte recruitment and systemic inflammation. Our observations highlight the possibility that sub-lineage specific differential systemic inflammation may be an explaination for the sub-lineage differences observed in the prevalence of neurological sysmptoms in humans. Based on our findings, it is worth investigating the early treatment of ZIKV infections in adults with anti-inflammatory medication to prevent possible ZIKV disease complications.

## Supplementary Material

TEMI-2021-0139_Supplementary_Figure.docx
