# Supplementary material for: Systemic inflammation, innate immunity and pathogenesis after Zika virus infection in cynomolgus macaques are modulated by strain-specificity within the Asian lineage
Source: Emerg Microbes Infect. 2021 Jun 14;10(1):1457–70. doi: 10.1080/22221751.2021.1943536 (PMC8300938; doi:10.1080/22221751.2021.1943536)
Supplement: TEMI-2021-0139_Supplementary_Figure.docx [file TEMI_A_1943536_SM8654.docx]

**Supplmentary Material**

**Supplmentary Figure 1. Correlation analysis of ZIKV RNA titers, cytokine and immune cell parameters.**

The correlation of the AUC of measured paramenters were compared against each other. Spearman rank-order correlation coefficients greater than 0 are represented in blue circles and those less than 0 are represented as red circles. Only statistically significant (i.e. *P*<0.05) Spearman correlations are represent in circles on the above graph.
